# Older Adults’ Experiences of Commercial Virtual Reality for Stroke Rehabilitation: A Mixed-Methods Study

**DOI:** 10.3390/medicina62030577

**Published:** 2026-03-19

**Authors:** Minjoon Kim, Chirathip Thawisuk, Shunichi Uetake, Hyeong-Dong Kim

**Affiliations:** 1 Department of Physical Therapy, School of Health and Environmental Science, College of Health Science, Korea University, Seoul 02841, Republic of Korea; ehrbs70@naver.com; 2 Department of Rehabilitation, Reiwa Rehabilitation Hospital, Chiba 260-0026, Japan; 3 Department of Physical Therapy, Graduate School of Human Health Sciences, Tokyo Metropolitan University, Tokyo 116-8551, Japan; 4 Department of Occupational Therapy, Faculty of Associated Medical Sciences, Chiang Mai University, Chiang Mai 50200, Thailand; chirathip.t@cmu.ac.th; 5 Department of Occupational Therapy, Graduate School of Human Health Sciences, Tokyo Metropolitan University, Tokyo 116-8551, Japan; 6 Department of Physical Therapy, Touto Rehabilitation College, Tokyo 153-0044, Japan; uetake@toutoreha.ac.jp; 7 Department of Health Science, School of Health and Environmental Science, College of Health Science, Korea University, Seoul 02841, Republic of Korea

**Keywords:** stroke, older adults, virtual reality, rehabilitation, patient experience, technology acceptance, simulator sickness

## Abstract

*Background and Objectives*: Stroke is a leading cause of long-term disability in older adults, who often face persistent motor, cognitive, and functional challenges. Conventional stroke rehabilitation programs often involve highly repetitive motor tasks, which may reduce patient motivation and contribute to suboptimal adherence over time. Virtual reality (VR) offers an engaging alternative; however, much of the existing research has focused on specialized rehabilitation-oriented VR systems rather than off-the-shelf commercial platforms. This study evaluated older stroke survivors’ acceptance, tolerability, and lived experiences of a short VR-based rehabilitation session using a commercial game on a commercial wearable VR system. *Methods*: A single-session convergent mixed-methods design was employed. Thirteen community-dwelling older stroke survivors (mean age 79.2 ± 7.1 years; 9 males, 4 female) completed a 15 min VR session using a commercial wearable VR system. The Technology Acceptance Model (TAM) questionnaire and Simulator Sickness Questionnaire (SSQ) assessed acceptance and tolerability, while semi-structured interviews explored lived experiences. Qualitative data were thematically analyzed. *Results*: Participants reported high acceptance across all TAM domains (overall M = 4.35 ± 0.79, scale 1–5). Enjoyment/intention to use was rated highest (M = 4.77 ± 0.42), while perceived usefulness was lowest (M = 4.15 ± 0.77). VR was well tolerated: the SSQ total score was 17.38 ± 1.73, with most symptoms rated at the mild level only. Exploratory Spearman correlations revealed a significant positive association between age and SSQ total score (r_h_ = +0.568, *p* = 0.043). Thematic analysis identified five themes: (1) usability and accessibility; (2) therapeutic value; (3) engagement and motivation; (4) social and clinical support; and (5) physical and cognitive demands. *Conclusions*: A commercial wearable VR system was found to be acceptable, safe, and engaging for older stroke survivors. With supervision and therapeutic framing, it may serve as a motivating adjunct to conventional rehabilitation.

## 1. Introduction

Stroke remains one of the leading causes of death and long-term disability worldwide, with an estimated 12.2 million new cases occurring annually [1]. Advances in acute medical management have improved survival rates, but a growing number of stroke survivors now live with persistent physical, cognitive, and psychosocial impairments [2]. Upper and lower limb dysfunction, reduced balance, and difficulties with attention or concentration are common sequelae, particularly due to motor and functional impairments including paresis or paralysis of the upper and lower limbs, often resulting in activity limitations and restrictions in participation [3,4]. Effective rehabilitation is therefore critical to promoting recovery and enhancing quality of life. However, despite strong evidence for rehabilitation efficacy, adherence to therapy programs is often limited by their repetitive nature, physical demands, and declining motivation over time, particularly in older adults [5,6]. Sustaining patient engagement remains one of the most pressing challenges in stroke rehabilitation [6]. Previous research indicates that adherence to post-stroke exercise programs varies considerably; in controlled settings, intervention completion rates range from 65% to 100% [7]. However, adherence in routine clinical populations may be lower, with one study reporting a mean rehabilitation exercise adherence rate of 68.6% among acute stroke patients [8], indicating only moderate engagement with prescribed rehabilitation programs.

Virtual reality (VR) has emerged as a promising adjunct to conventional therapy, offering interactive, immersive, and game-like environments that can increase motivation while supporting motor and cognitive recovery [9]. In recent years, VR technologies have been increasingly adopted across medical fields including rehabilitation due to their ability to simulate controlled therapeutic environments and provide multisensory feedback to users [10]. Systematic reviews and meta-analyses suggest that VR-based interventions can enhance upper and lower limb function, balance, and participation, while also improving treatment adherence compared to traditional rehabilitation approaches [11,12,13]. Immersive VR delivered via head-mounted displays provides a sense of presence and enjoyment, both of which are key drivers of active participation in rehabilitation tasks [14].

From a physiological perspective, VR-based training may facilitate neuroplasticity by promoting task-specific, repetitive motor practice that supports cortical reorganization and sensorimotor reweighting [15,16]. Moreover, VR allows for the delivery of repetitive, task-oriented training with real-time feedback, aligning with principles of neuroplasticity [16]. Real-time visual feedback and augmented error signals can enhance motor learning processes by reinforcing correct movement patterns and supporting error correction during practice [17]. Biomechanically, VR tasks often involve repetitive, task-oriented upper-limb movements and rapid stimulus–response actions that promote coordination, dexterity, and motor control, stimulating neural circuits involved in post-stroke motor recovery [18]. Nevertheless, tolerability issues such as cybersickness, visual strain, and cognitive fatigue remain concerns, particularly for older survivors who may be more vulnerable to sensory overload [19].

Most prior research has focused on VR systems specifically designed for rehabilitation [20]. While these systems can be highly effective, their adoption in everyday practice is limited by high costs, specialized training requirements, and restricted availability in many healthcare settings [21,22]. In contrast, commercially available VR platforms are relatively affordable, portable, and increasingly accessible. These systems feature a wide variety of interactive games that, although not originally developed for medical use, can be repurposed for therapeutic purposes such as promoting movement, reaction time, coordination, and engagement [14]. Their ease of setup and intuitive user interfaces may reduce barriers for both clinicians and patients, making them a potentially valuable tool for broadening access to engaging rehabilitation experiences [23]. For the present study, the commercial exergame MOVE FAST was selected because its core task demands rapid reaction time, upper-limb reaching, and stimulus–response mapping align with established principles of neuroplasticity-based motor training, specifically task-specific repetitive practice and attentional dual-task engagement [16].

Despite this potential, little is known about how stroke survivors themselves perceive the use of commercial VR in rehabilitation. Research to date has primarily examined effectiveness and feasibility from clinical perspectives, while patient acceptance, tolerability, and lived experiences remain underexplored [20,24]. Yet, these perspectives are critical, as patient motivation and willingness to engage are strong predictors of adherence and therapeutic success [25,26]. Understanding whether stroke survivors find commercial VR games acceptable, tolerable, and meaningful to their rehabilitation can help inform safe, patient-centered integration into clinical practice.

The purpose of this study was to evaluate stroke survivors’ acceptance, tolerability, and experiences of a short VR-based rehabilitation session using a commercial game on a commercial wearable VR system. By combining quantitative measures of technology acceptance and simulator sickness with qualitative accounts of lived experiences, this study provides insights into the feasibility and clinical relevance of using commercially available VR platforms as an adjunct to conventional stroke rehabilitation.

## 2. Materials and Methods

### 2.1. Design and Setting

We conducted a convergent mixed-methods study in a rehabilitation setting, following the approach outlined by Creswell and Clark [27]. Quantitative data (questionnaires) and qualitative data (semi-structured interviews) were collected during the same encounter, analyzed independently, and then integrated during interpretation to provide a comprehensive understanding of participants’ acceptance, tolerability, and experiences.

### 2.2. Participants

A convenience sample of 13 adult stroke survivors receiving rehabilitation services was recruited during June 2025. Inclusion criteria were: age ≥ 65 years, a clinical diagnosis of ischemic or hemorrhagic stroke in the subacute or chronic phase, medical stability to tolerate brief headset use in sitting, and sufficient cognitive ability to communicate and follow instructions (Mini-Mental State Examination [MMSE] ≥ 24). Exclusion criteria included uncontrolled vestibular disorders or severe motion sickness, active epilepsy, severe uncorrected visual or hearing impairments that would preclude VR use, or any condition deemed unsafe for headset exposure. All participants provided written informed consent. The study was approved by the institutional ethics committee and conducted in accordance with the Declaration of Helsinki.

### 2.3. VR Exposure: MOVE FAST on a Commercial Wearable VR System (Meta Quest 3, Meta Platforms Inc., Menlo Park, CA, USA)

The commercial VR exergame MOVE FAST on the commercial wearable VR system was selected for its simplicity of interaction and therapeutic relevance to stroke rehabilitation. As a reaction-time-oriented game, it requires rapid stimulus–response mapping and upper-limb reaching, movements directly related to motor recovery and attentional training. Its color-coded targets and controller inputs offer an accessible interface for older adults while maintaining challenge through speed and accuracy demands. Gameplay can also be delivered in a seated, fall-safe position, reducing risk while supporting active engagement. This choice aligns with recommendations for brief, supervised VR exposure in older adults to minimize cybersickness and sensory overload while fostering positive first experiences, rather than relying on specialized medical VR systems with limited accessibility [14].

### 2.4. Measures

#### 2.4.1. Technology Acceptance Model Questionnaire

To evaluate participants’ acceptance of VR rehabilitation, we employed a brief questionnaire adapted from the Technology Acceptance Model (TAM), which has been widely used to assess adoption of new technologies in healthcare [28]. TAM was chosen because it captures key determinants of technology use—perceived ease of use, perceived usefulness, social influence, and behavioral intention—which are particularly relevant to understanding older adults’ willingness to engage with VR [28]. All items were rated on a 5-point Likert scale (1 = strongly disagree to 5 = strongly agree), and domain scores were calculated as item-level means. The questionnaire was adapted from Shintaro and Tetsuaki [29] and underwent content validity testing using the Index of Item-Objective Congruence (IOC), with all items scoring above 0.5.

#### 2.4.2. Simulator Sickness Questionnaire

Immediately after the VR session, participants completed the Simulator Sickness Questionnaire (SSQ) to capture the presence and severity of cybersickness-related symptoms. Each symptom was rated as none, mild, moderate, or severe. Symptoms assessed included general discomfort, headache, nausea, eyestrain, difficulty focusing, dizziness/vertigo (with eyes open or closed), imbalance, sweating, stomach awareness, and burping [30].

#### 2.4.3. Semi-Structured Interviews

After completing the questionnaires, participants took part in a 15–20 min semi-structured interview informed by the TAM. The guide explored initial impressions and usability, perceived motor, cognitive, and psychosocial value, discomfort and challenges related to game mechanics, social and professional support, and preferences for future sessions (e.g., duration, difficulty, or content). All interviews were audio-recorded. The protocol was piloted prior to data collection with three older adults who were not part of the study population, to refine clarity and flow.

### 2.5. Procedure

After eligibility was confirmed and written informed consent was obtained, participants completed a brief pre-session safety check, which included verification of seating stability, headset fit, and comfort with controller use. A standardized onboarding lasting 1–2 min was then provided, covering the purpose of the activity, safety instructions, controller inputs, and an opportunity for brief acclimatization to the VR environment.

Each participant performed a single 15 min session of the MOVE FAST exergame on the commercial wearable VR system in a seated, fall-safe setup (a stable chair with armrests and a cleared perimeter). A therapist supervised all sessions and could provide cueing, pause the system, or terminate the session at any sign of distress or fatigue. Immediately following gameplay, participants completed the TAM questionnaire and SSQ, and then participated in a semi-structured interview.

**Figure 1:**
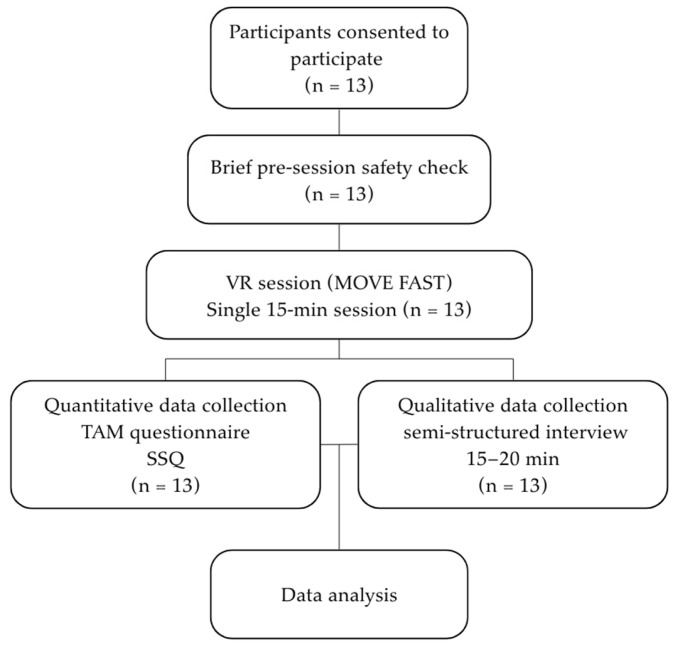
Overview of study process.

All sessions were conducted by the same therapist to ensure procedural consistency. Standard safety monitoring, including observation for dizziness, imbalance, or discomfort, was in place throughout the procedure to minimize risk and ensure participant well-being (Figure 1).

### 2.6. Data Analysis

Quantitative data were analyzed using Microsoft Excel. Descriptive statistics (means, ranges, standard deviations, and percentages) were used to summarize the data. Exploratory associations between variables were examined using Spearman’s rank correlation coefficient, which is appropriate for small sample sizes and non-parametric data distributions [31].

Interview transcripts were analyzed using reflexive thematic analysis with a hybrid deductive–inductive strategy [32]. Segments were first mapped to TAM domains and tolerability constructs then inductively organized into subthemes (e.g., onboarding/pacing, feedback clarity, content variety, cognitive engagement, safety/supervision). Coding was conducted independently by the first author (MK) and third author (SU), with discrepancies resolved through discussion and consensus. Themes were further reviewed with the wider research group. Data management was supported using ALASti version 25.0.1.

Mixed-methods integration followed a connecting and contiguous approach [33]. Quantitative findings guided participant sampling for the qualitative phase, and final integration occurred at the interpretation stage by aligning quantitative patterns with qualitative explanations.

## 3. Results

### 3.1. Quantitative Results

#### 3.1.1. Demographic Data

The participants included 9 males and 4 females with diagnoses of either hemorrhagic (*n* = 9) or ischemic (*n* = 4) stroke. Participant ages ranged from 70 to 94 years, with a mean age of 79.2 years (SD = 7.1). The majority of participants (*n* = 11) had a high school level of education, while one participant had an advanced diploma and another had a bachelor’s degree. Cognitive function, as assessed by the MMSE, showed scores ranging from 24 to 30 (M = 27.2, SD = 3.1). Functional independence, measured by the Functional Independence Measure (FIM), varied from 53 to 125 among the participants (Table 1).

#### 3.1.2. Acceptance of VR

Overall, participants reported high acceptance of VR, with mean scores ranging from 4.08 to 4.77. Perceived ease of use was rated highly, particularly for ease of understanding (M = 4.54) and helpfulness in therapy (M = 4.46). Perceived usefulness received a moderate score (M = 4.15). Social influence was positively endorsed, with support from family and friends (M = 4.31) and healthcare teams (M = 4.38). The highest rating was for intention to use, with participants finding VR therapy enjoyable (M = 4.77). Under behavioral intention, participants planned to continue using VR (M = 4.38), though willingness declined slightly when alternatives were available (M = 4.08) or when effort was required (M = 4.15) (Table 2).

#### 3.1.3. Simulator Sickness

Overall, symptoms were mostly reported at a mild level, with very few moderate or severe cases. The most frequently reported mild symptoms were general discomfort (100%), headache (100%), and fatigue (77%). A small number of participants experienced moderate symptoms, most notably difficulty concentrating (*n* = 4), followed by fatigue (*n* = 2), eyestrain (*n* = 2), increased salivation (*n* = 2), sweating (*n* = 2), and blurred vision (*n* = 2). Severe symptoms were rare, with only one participant each reporting severe fatigue and severe difficulty focusing. Importantly, no participants reported nausea, vertigo, or dizziness beyond mild levels (Table 3).

#### 3.1.4. Correlation Analyses

Spearman rank correlation analyses were conducted to examine whether age or cognitive status (MMSE) was associated with each of the five TAM domains (perceived ease of use], perceived usefulness [PU], Social Influence [SI], Intention to Use [IU], Behavioral Intention [BI]) and SSQ total score (*n* = 13). Full results are presented in Table 4.

Age was not significantly correlated with PEOU (r_h_ = +0.082, *p* = 0.790), PU (r_h_ = −0.015, *p* = 0.962), SI (r_h_ = +0.404, *p* = 0.171), IU (r_h_ = −0.049, *p* = 0.873), or BI (r_h_ = +0.119, *p* = 0.699). However, age was significantly positively correlated with SSQ total score (r_h_ = +0.568, *p* = 0.043), indicating that older participants tended to report higher overall simulator sickness burden. MMSE was not significantly correlated with any TAM domain (PEOU: r_h_ = +0.364, *p* = 0.222; PU: r_h_ = −0.072, *p* = 0.816; SI: r_h_ = +0.074, *p* = 0.810; IU: r_h_ = −0.025, *p* = 0.936; BI: r_h_ = −0.286, *p* = 0.344) or with SSQ total (r_h_ = +0.052, *p* = 0.867). These findings should be interpreted cautiously given the small sample size (*n* = 13) and the exploratory nature of the analyses. Nevertheless, the significant age–SSQ association suggests that age-stratified monitoring and adaptive session protocols may be warranted in future VR rehabilitation trials.

### 3.2. Qualitative Results

Analysis of the interviews yielded five key themes describing stroke survivors’ experiences of using virtual reality (VR) in rehabilitation (Table 5). These themes reflected perspectives on VR usability, perceived clinical value, engagement and motivation, social and clinical support, and physical and cognitive demands.

#### 3.2.1. Perceived Usability and Accessibility

Many participants described the VR system as straightforward and manageable, even for those with no prior experience using digital tools. Positive impressions of usability included both operational ease and clarity of instructions.

“It was easy to use. The instructions were simple enough, and I didn’t have to make a huge effort to understand what to do. I could follow along without any confusion.”

(P3)

“I gave it a five on the questionnaire because I really thought it was easy. I didn’t feel confused or worried during the experience. I just did what I was told, and it worked fine.”

(P4)

However, for others—especially those unfamiliar with digital interfaces—the experience involved a degree of initial disorientation. In these cases, unfamiliarity with VR elements like colour-coded signals or motion stimuli created uncertainty.

“I wasn’t sure what to expect because it was my first time. The colours showed up, and I didn’t know what they meant at first. I got used to it after a while, but in the beginning, I felt a little lost.”

(P10)

Several older participants directly attributed their hesitation or difficulty to their age or lack of technological familiarity, suggesting a potential need for more comprehensive pre-use orientation.

“It was a bit difficult for me to follow, probably because of my age. I’ve never used anything like this before, and it’s not easy to understand something completely new at this stage in life.”

(P9)

#### 3.2.2. Therapeutic Value and Clinical Perception

Participants expressed diverse perspectives on whether VR contributed meaningfully to their physical rehabilitation. Some reported a clear sense that the VR exercises had therapeutic benefits—particularly in areas such as upper limb mobility or reflex training.

“I think it will help in the future, especially with how I move my arms. It’s not just playing a game—it’s real movement that requires coordination. That’s important for rehab, I think.”

(P11)

Others valued the feedback mechanisms offered by VR, which they viewed as motivating and objective.

“Being able to see the numbers and the feedback was helpful. In regular rehab, it’s not always clear how well you’re doing, but this gave me something I could understand directly.”

(P1)

On the other hand, several participants were unsure whether VR had any direct therapeutic benefit. Their uncertainty stemmed from either the simplicity of the tasks or their lack of understanding of the connection between the activity and their recovery.

“I guess it was okay, maybe a four out of five. But honestly, I don’t know how useful it was. It didn’t feel like real therapy. It was just punching at things, like a game.”

(P3)

“I don’t think this can really help with daily life skills. It’s too different from what I actually need to do every day.”

(P9)

#### 3.2.3. Engagement, Motivation, and Enjoyment

A widely shared positive experience among participants was the enjoyable and stimulating nature of VR-based therapy. Several commented on the motivational value of the sessions, which contrasted with the monotony they sometimes felt in conventional rehabilitation.

“It was fun, really. I don’t usually look forward to rehab, but with this, I kind of wanted to try it again. It felt like something different—not just doing exercises, but playing while moving. That makes a big difference.”

(P5)

“It was a fun kind of therapy. I wasn’t just sitting there doing the usual things; I had to focus and respond. I liked that part. I think if more therapies were like this, I’d probably want to keep going.”

(P10)

However, not all participants were equally enthusiastic. Some were open to continued use under certain conditions, such as having no better alternatives or needing to see more results.

“If I had the opportunity, I’d like to do it again. But I’m not sure I’d choose it over other methods unless it was really shown to be better. I guess it depends on what options I have.”

(P4)

“I didn’t dislike it, but I also didn’t feel strongly that I wanted to do it again. It was just okay.”

(P11)

#### 3.2.4. Social and Clinical Support

Support from family, friends, and the clinical team was described as uncertain or hypothetical by many participants. Few had explicitly discussed VR therapy with others, so their views reflected assumptions about how others might respond.

“I haven’t really talked to my family about it, but I think they would be supportive. They usually are. I imagine they’d say something like ‘give it a try.’”

(P4)

“I think the rehab staff would recommend it. They seemed positive about it when they explained it to me, though it wasn’t a strong push either way.”

(P2)

Others were less confident, particularly when considering whether medical staff would endorse the approach.

“I don’t think my doctor or therapists would really recommend this. It seems too new, too experimental. They didn’t say much, so maybe they’re not sure either.”

(P11)

#### 3.2.5. Physical and Cognitive Demands

While engagement was generally high, many participants also described physical or cognitive strain associated with VR therapy. This included fatigue, dizziness, difficulty concentrating, or challenges with visual focus.

“It was hard to concentrate at times. The things moved quickly on the screen, and I had to react fast. That was a bit stressful, even though it was just a simulation.”

(P5)

“The colors and shapes came quickly, and I couldn’t always tell what to do. My reflexes aren’t what they used to be, so it was harder for me to keep up.”

(P11)

“I had trouble seeing things clearly. My vision isn’t great anymore, and with the headset on, some parts looked blurry or hard to focus on.”

(P9)

Yet not all participants viewed these difficulties negatively. Some regarded the challenge itself as therapeutic, particularly in stimulating reflexes and cognitive attention.

“I think it helped with my reaction time. I had to think and move quickly, which I don’t usually do anymore. That’s good practice for me.”

(P1)

Participants also highlighted the need for adaptability in the system—specifically, being able to customize elements such as task speed or difficulty level.

“Some parts were too fast, but I think that depends on the person. The system should adjust based on the user, because everyone’s reaction time is different.”

(P1)

## 4. Discussion

This study evaluated stroke survivors’ acceptance, tolerability, and lived experiences of a short VR-based rehabilitation session using a commercial game on Meta Quest. The findings indicated high acceptance and enjoyment, with tolerability characterized by mostly mild simulator sickness symptoms. At the same time, perceptions of therapeutic benefit varied, underscoring VR’s potential as an adjunct to conventional stroke rehabilitation while also revealing barriers such as uncertainty about clinical value and long-term use.

Participants reported high acceptance across TAM domains, with enjoyment rated highest. The engaging and motivating qualities of VR align with previous reviews that emphasize VR’s ability to sustain motivation, enhance adherence, and counteract the monotony of conventional therapy [10,11]. Notably, even older participants with limited digital experience found VR easy to use, supporting evidence that simple, intuitive interfaces make VR feasible across diverse populations [26]. This highlights the accessibility of commercial VR platforms for rehabilitation contexts, where ease of use can reduce training demands on both patients and therapists [34]. However, perceived usefulness was only moderate. Several participants questioned whether the VR game directly supported daily function, echoing prior meta-syntheses showing that patients often struggle to link virtual exercises to real-life rehabilitation goals [25]. This finding underscores the importance of therapists framing VR sessions within functional and task-related goals.

The VR session was generally well tolerated, with only mild symptoms such as fatigue, eyestrain, and difficulty concentrating. No participants reported nausea or vertigo, which are often the most prohibitive side effects of immersive VR. These results align with pilot studies demonstrating that VR can be delivered safely in stroke populations with high satisfaction and minimal adverse events [35]. Nonetheless, reports of moderate concentration difficulty point to the need for tailored pacing and careful selection of game intensity. In clinical practice, this suggests that therapists should introduce VR in short, structured sessions, monitor symptoms closely, and provide breaks to ensure safety [36,37]. For older adults, who may be more vulnerable to disorientation and cognitive strain [19], additional orientation and supervision are particularly important.

Exploratory Spearman correlation analysis revealed a significant positive association between age and SSQ total score (r_h_ = +0.568, *p* = 0.043), suggesting that older participants tended to experience greater simulator sickness during the VR session. This finding aligns with previous research indicating that age-related declines in vestibular function, visual acuity, and sensorimotor integration may increase susceptibility to cybersickness due to greater difficulty reconciling sensory conflicts between visual and vestibular cues [19]. Consistent with concerns raised in the qualitative findings regarding physical and cognitive demands, immersive VR may therefore place greater strain on older participants. Clinically, this suggests the need for age-sensitive VR implementation strategies, such as shorter sessions, slower visual flow, and simplified visual environments [38]. However, given the exploratory nature of this analysis and the small sample size, these findings should be interpreted cautiously and require confirmation in larger studies.

Qualitative findings revealed strong enthusiasm for VR’s enjoyable and game-like qualities. Many participants contrasted VR favorably with conventional therapy, echoing systematic reviews that highlight VR’s ability to enhance motivation and provide a sense of presence absent from traditional rehabilitation [13,14]. This novelty-driven engagement may be especially valuable in sustaining adherence during repetitive rehabilitation routines, particularly in community or outpatient settings where maintaining motivation can be challenging [39]. However, ambivalence about therapeutic value persisted among some participants. A number perceived VR as “just a game,” a theme consistent with qualitative syntheses where patients valued novelty but doubted clinical efficacy [25,40]. This finding highlights the need for therapists to explicitly link commercial game activities such as reaching, punching, or reacting to stimuli to underlying motor and cognitive rehabilitation goals [41,42]. Without this framing, patients may fail to appreciate VR’s therapeutic relevance, limiting its effectiveness in practice.

Participants anticipated general support from family and clinicians but were uncertain about strong professional endorsement. Prior studies confirm that patient confidence in VR is shaped by legitimacy and encouragement from healthcare teams [25]. In our study, the absence of explicit clinician endorsement contributed to participants’ uncertainty about VR’s status as a therapeutic tool. Adoption is facilitated when therapists present VR not as a novelty but as a validated adjunct integrated into rehabilitation programs [10]. This finding underscores the critical role of clinicians in legitimizing VR use, guiding expectations, and embedding commercial systems into structured therapy rather than treating them as entertainment add-ons [42].

While participants reported enjoyment, they also described cognitive strain, difficulty focusing, and visual challenges. Interestingly, some reframed these demands as therapeutic opportunities for reflex training and attentional practice. In practice, these findings suggest that commercial VR games can serve dual purposes: providing enjoyable engagement while also offering implicit cognitive and motor challenges [23]. However, since task demands are not tailored to rehabilitation, clinicians must carefully select which commercial games to use, balancing stimulation with safety [42]. Games with simple rules, slower pacing, and clear visual feedback may be most appropriate for older stroke survivors [20,23], whereas fast or visually complex games may risk frustration or fatigue [20].

These findings support the feasibility of integrating commercially available VR games into stroke rehabilitation as an adjunct to conventional therapy. To maximize benefit, therapists should: (a) provide orientation and supervision during sessions, (b) select commercial games with therapeutic potential (e.g., those requiring reaching, balance, or reaction), (c) adapt session length and intensity to patient tolerance, and (d) frame gameplay in terms of rehabilitation goals to reinforce clinical relevance. These strategies are consistent with recommendations from meta-reviews advocating for patient-centered and goal-oriented VR implementation [11,37].

From a physiological perspective, VR may promote neuroplasticity through task-specific, repetitive motor practice that supports cortical reorganization and sensorimotor reweighting [16]. Real-time visual feedback provides augmented error signals that facilitate motor learning processes. Biomechanically, the upper-limb reaching and rapid stimulus–response demands of MOVE FAST may activate corticospinal and cerebellar pathways involved in post-stroke motor recovery. Psychologically, the immersive game environment may induce a flow state, enhancing engagement and intrinsic motivation, an important factor given the challenge of sustaining participation in stroke rehabilitation [39]. Thus, the potential benefits of VR likely arise from the combined neurological, biomechanical, and psychological mechanisms that support rehabilitation outcomes.

Poor adherence to stroke rehabilitation is multifactorial. In addition to the repetitive nature of conventional therapy, structural barriers such as transportation difficulties, caregiver burden, financial constraints, and limited access to rehabilitation facilities often contribute to dropout, particularly among older adults [5,6]. Commercial VR platforms may help address some of these barriers through portability and the potential for home-based use. However, VR also introduces new challenges, including device cost, digital literacy requirements, and the need for adequate physical space. To improve tolerability for older adults, clinicians may consider shorter initial sessions (10–15 min), slower game speeds, and gradual progression of difficulty, while developers could incorporate adaptive difficulty settings and improved visual contrast [38,43].

This study was designed as a feasibility assessment rather than an efficacy trial, consistent with the feasibility-to-efficacy framework in rehabilitation research [44]. Establishing safety, user acceptability, and measurement suitability is an essential precursor to comparative intervention trials. Having addressed these prerequisites, future research should proceed to randomized controlled trials comparing VR-based and conventional rehabilitation using larger samples and functional outcome measures.

Several limitations should be acknowledged. The single-session design may be influenced by novelty effects, which can inflate technology acceptance ratings [26]. Longitudinal studies are therefore needed to evaluate sustained engagement. The small sample size (n = 13), single-site recruitment in Japan, and relatively homogeneous participant characteristics limit statistical power and generalizability. Additionally, clinical variables such as stroke chronicity, BMI, medications, and comorbidities were not collected and should be included in future studies. Finally, the commercial VR game used was not specifically designed for rehabilitation, which may have influenced participants’ perceptions of its therapeutic value. Future adequately powered trials will be needed to determine comparative effectiveness.

Future studies should evaluate the integration of commercial VR systems into repeated rehabilitation sessions and assess their impact on functional recovery, motivation, and quality of life. Research should also explore strategies for therapist-led selection and adaptation of commercial games, examining which genres or task demands align most effectively with rehabilitation goals. Including clinician perspectives will be essential to address practical considerations such as session planning, safety monitoring, and patient education.

## 5. Conclusions

A commercially available, consumer-grade wearable VR system was found to be acceptable, safe, and engaging for older stroke survivors. With appropriate supervision and therapeutic framing, it may serve as a motivating adjunct to conventional rehabilitation. Clinicians play a critical role in legitimizing VR use, selecting appropriate games, and ensuring sessions are adapted to individual patient needs.

## Figures and Tables

**Table 1:** Demographic data of participants.

Participant	Sex	Diagnosis	Age	Level of Education	MMSE	FIM
P1	Man	Hemorrhagic	74	Advanced Diploma	30	118
P2	Man	Hemorrhagic	94	Bachelor	30	93
P3	Man	Ischemic	71	High School	30	116
P4	Woman	Hemorrhagic	81	High School	29	96
P5	Man	Hemorrhagic	82	High School	29	71
P6	Man	Ischemic	74	High School	30	125
P7	Man	Hemorrhagic	90	High School	24	74
P8	Man	Hemorrhagic	81	High School	25	53
P9	Woman	Ischemic	76	High School	29	122
P10	Man	Hemorrhagic	81	High School	25	53
P11	Woman	Hemorrhagic	70	High School	28	105
P12	Woman	Hemorrhagic	71	High School	26	80
P13	Man	Ischemic	84	High School	28	98

**Table 2:** Average scores of TAM questionnaire items and domain averages (*n* = 13).

Domain	Item	M	SD
Perceived Ease of Use	VR is easy to understand and easy to use.	4.54	0.75
	VR can be mastered without much effort.	4.23	0.97
	VR helps in therapy.	4.46	0.50
	Domain average	4.41	0.78
Perceived Usefulness	VR improves performance in daily living activities.	4.15	0.77
Social Influence	Family/friends support VR use.	4.31	0.91
	Healthcare team recommends VR.	4.38	0.84
	Domain average	4.35	0.88
Intention to Use	VR therapy is enjoyable.	4.77	0.42
Behavioral Intention	I plan to continue using VR in therapy.	4.38	0.84
	I will continue to use VR even if other options are available.	4.08	0.83
	I will continue to use VR in therapy even if it requires effort.	4.15	0.66
	Domain average	4.21	0.79

**Table 3:** Simulator Sickness Questionnaire results (*n* = 13).

Symptom	No Symptom	Mild	Moderate	Severe
General discomfort	-	13	-	-
Fatigue	-	10	2	1
Headache	-	13	-	-
Eyestrain	-	11	2	-
Difficulty focusing	-	12	-	1
Increased salivation	-	11	2	-
Sweating	-	11	2	-
Nausea	-	13	-	-
Difficulty concentrating	-	9	4	-
Fullness of head	-	13	-	-
Blurred vision	-	11	2	-
Dizziness (eyes open)	-	13	-	-
Dizziness (eyes closed)	-	13	-	-
Vertigo	-	13	-	-
Stomach awareness	-	13	-	-
Burping	-	13	-	-

**Table 4:** Exploratory Spearman rank correlations between participant characteristics and VR outcomes (*n* = 13).

Predictor	Outcome	r_s_	*p*-Value
Age (years)	PEOU	0.082	0.790
	PU	−0.015	0.962
	SI	0.404	0.171
	IU	−0.049	0.873
	BI	0.119	0.699
	SSQ	0.568 *	0.043 *
MMSE score	PEOU	0.364	0.222
	PU	−0.072	0.816
	SI	0.074	0.810
	IU	−0.025	0.936
	BI	−0.286	0.344
	SSQ	+0.052	0.867

* indicate the significant result.

**Table 5:** Themes arising from qualitative analysis.

Theme	Sub-Theme
1. Perceived Usability and Accessibility	Ease of Use and Interface Clarity
	First-Time Confusion and Learning Curve
	Age-Related Accessibility Barriers
2. Therapeutic Value and Clinical Perception	Perceived Contribution to Physical Recovery
	Usefulness of Measurable Feedback
	Skepticism Toward Clinical Impact
3. Engagement, Motivation, and Enjoyment	Enjoyment and Positive Emotional Response
	Increased Willingness to Participate in Rehabilitation
	Conditional or Moderate Motivation
4. Social and Clinical Support	Perceived Support from Family or Friends
	Ambiguity in Clinical Endorsement
5. Physical and Cognitive Demands	Sensory and Cognitive Load
	Positive Challenge and Cognitive Stimulation
	Need for Individualised Calibration

## Data Availability

The data that support the findings of this study are available from the authors upon reasonable request.

## Abbreviations

The following abbreviations are used in this manuscript:

FIM	Functional Independence Measure
IOC	Index of Item-Objective Congruence
MMSE	Mini-Mental State Examination
SSQ	Simulator Sickness Questionnaire
TAM	Technology Acceptance Model
VR	Virtual Reality
PEOU	Perceived Ease of Use
PU	Perceived Usefulness
SI	Social Influence
IU	Intention to Use
BI	Behavioral Intention
